# A Review of Femtosecond Laser Processing of Silicon Carbide

**DOI:** 10.3390/mi15050639

**Published:** 2024-05-10

**Authors:** Quanjing Wang, Ru Zhang, Qingkui Chen, Ran Duan

**Affiliations:** School of Mechanical and Electronic Engineering, Shandong Jianzhu University, Jinan 250101, China; wqj338@163.com (Q.W.); cqk@sdjzu.edu.cn (Q.C.); duanran20@sdjzu.edu.cn (R.D.)

**Keywords:** femtosecond laser, silicon carbide, processing, application

## Abstract

Silicon carbide (SiC) is a promising semiconductor material as well as a challenging material to machine, owing to its unique characteristics including high hardness, superior thermal conductivity, and chemical inertness. The ultrafast nature of femtosecond lasers enables precise and controlled material removal and modification, making them ideal for SiC processing. In this review, we aim to provide an overview of the process properties, progress, and applications by discussing the various methodologies involved in femtosecond laser processing of SiC. These methodologies encompass direct processing, composite processing, modification of the processing environment, beam shaping, etc. In addition, we have explored the myriad applications that arise from applying femtosecond laser processing to SiC. Furthermore, we highlight recent advancements, challenges, and future prospects in the field. This review provides as an important direction for exploring the progress of femtosecond laser micro/nano processing, in order to discuss the diversity of processes used for manufacturing SiC devices.

## 1. Introduction

SiC has gained significant attention as a promising material for various applications due to its exceptional properties, including high hardness, excellent thermal conductivity, and chemical inertness [1]. Compared to silicon, SiC has many advantages, including higher operating temperature, lower conduction loss, higher bandgap, and higher critical breakdown field strength. SiC is a widely utilized advanced material and a representative of third-generation wide bandgap semiconductor materials. SiC is essential for exploring its potential in diverse fields such as electronics, photonics, aerospace, and microelectromechanical systems (MEMS) [2,3,4].

**Figure 1:**
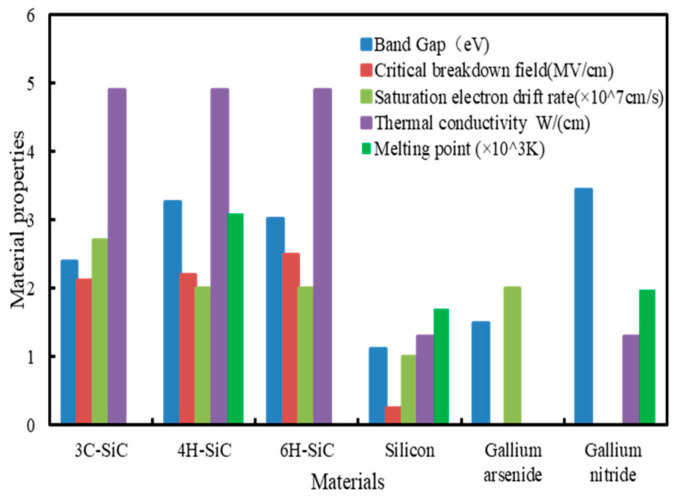
Comparison of material properties.

SiC exists in the forms of single crystal, polycrystalline, and amorphous [5]. At present, over 250 types of allotrope crystals of single crystal SiC have been discovered [6]. Figure 1 shows the semiconductor properties of several common single crystals SiC and other semiconductor materials [7,8,9]. The band gap of SiC is higher than that of silicon and Gallium arsenide, slightly lower than that of Gallium nitride, and the band gap of 4H-SiC is higher than that of 3C-SiC and 6H-SiC. Due to the characteristics of wide bandgap semiconductors, SiC exhibits high stability. The critical breakdown field of SiC is about 10 times that of silicon, the saturation electric drive rate of SiC is about 2 times that of silicon, the thermal conductivity of SiC is 3–4 times that of silicon and Gallium nitride, and the melting point of SiC is 1.5–1.8 times that of silicon and gallium nitride.

Other emerging materials, such as 2D semiconductor materials, have been investigated by many scholars. Antimonene, an emerging 2D semiconductor material, features a customizable bandgap, high carrier mobility, low thermal conductivity, and outstanding optical properties. These qualities make its interaction with photons highly promising in nanophotonic applications such as photodetectors, solar cells, photocatalysis, cancer treatment, surface plasmon resonance sensors, and nonlinear photon devices [10]. Graphdiyne displays environmentally friendly properties, increased chemical stability, a large specific surface area, a narrow bandgap, and high carrier mobility [11]. Compared to many other carbon materials, it has greater potential in nanophotonic applications. Its photoelectric performance can be enhanced through element doping [12] and hybridization with other nanostructures [13]. Due to its exceptional photovoltaic performance, graphdiyne is utilized in solar cells [14]. Selenium nanostructures, as narrow bandgap semiconductors, have been synthesized with controllable size, shape, and structure. However, under harsh conditions, their stability is poor, and their electronic/optoelectronic performance is suboptimal. Therefore, doping or epitaxial growth techniques are commonly employed to introduce heteroatoms and enhance the performance of selenium nanomaterials [15]. Black phosphorus is a typical layered two-dimensional material with high carrier mobility, an in-plane anisotropic structure, and an adjustable direct bandgap. However, its sensitivity to the environment necessitates improvements in its physical and chemical properties through doping methods [16,17]. Bismuth possesses physical and chemical properties including a tunable bandgap, excellent photoresponse, strong diamagnetism, and high photothermal conversion efficiency. The synthesis of bismuth-based hybrid materials can be employed to create ultra-small and ultra-sensitive photodetectors, facilitate the photocatalytic degradation of organic pollutants, and advance biophotonics applications [18,19]. Two-dimensional tellurium exhibits environmental stability, a bandgap dependent on thickness, piezoelectric properties, high carrier mobility, and light responsiveness. Consequently, it finds application in photodetectors, energy harvesting devices, piezoelectric devices, and other areas [20]. To address issues such as surface redox reactions, inadequate specific capacity, and unstable output potential in water-based zinc-ion batteries, a novel zinc tellurium battery cathode material utilizing a conversion-type ion storage mechanism can be employed [21].

SiC is processed using various methods, with diamond wire saw cutting being a popular choice. However, SiC tends to experience crack damage during processing. Diamond wire saws may encounter challenges such as abrasive detachment, wire wear, surface blockage, and graphitization when utilized in SiC processing [22,23,24]. Ultra-precision polishing technology serves as the final step in the SiC machining process, employed to guarantee the surface roughness, flatness, and material removal rate of the SiC substrate [25,26]. Mechanical polishing is utilized to enhance the processing efficiency of polishing and improve the surface roughness of the substrate. Chemical mechanical polishing is employed to achieve ultra-smooth, defect-free, damage-free, and flat machined surfaces [27]. However, the ultra-precision polishing process of SiC single crystal substrates is complex and multifaceted, with the quality of polishing linked to the multi-dimensional motion trajectory of the polishing head. Consequently, further research is required to explore the polishing mechanism and process parameters. Yang et al. [28] studied ultrasonic-vibration-assisted electrochemical mechanical polishing to boost the effectiveness of conventional methods. This technique increased the contact force between abrasives and the SiC surface. Nevertheless, this method tends to raise the surface roughness of SiC. Wet etching technology comprises conventional wet etching and electrochemical etching. Conventional wet chemical etching of SiC typically involves high temperatures and is usually conducted in an alkaline solution of 488 K phosphoric acid or 373 K or above K_3_Fe(CN)_6_. Electrochemical etching is intricate, exhibiting low etching efficiency and poor uniformity [29]. Dry etching methods such as reactive ion etching are common in semiconductor processing [30]. However, the strong chemical stability of SiC requires a mask during dry etching, leading to low etching efficiency and a complicated process. Non-traditional, energy-assisted mechanical processing methods like vibration, laser, electrical, magnetic fields, chemical processes, advanced coolant systems, and others are employed for challenging materials such as hard and brittle substances [31,32,33]. These techniques improve machinability, enhance material removal rates, and boost surface quality. Laser processing can achieve efficient and high-precision machining of microstructures [34,35]. Zhao et al. proposed a novel method called laser-induced, oxidation-assisted milling to address surface damage issues commonly encountered in milling processes, such as pits, cracks, scratches, matrix coatings, and burrs [36,37]. In this method, an oxide layer is generated in the ablation zone through laser-induced oxidation, which is then easily removed by the milling tool. This process is repeated several times until the material is fully machined. Compared to conventional milling methods, no tool wear is observed in the milled oxide layer and the cutting forces are extremely low. Additionally, lower surface roughness can be achieved. However, laser processing can easily introduce thermal and physical damage.

Femtosecond laser technology has emerged as a powerful tool for the precise fabrication and modification of materials [38]. To achieve a uniform distribution of laser spot energy density, enable smaller aperture processing, and mitigate issues like edge collapse during processing, an attenuation module can be incorporated into the femtosecond laser precision micro-processing system [39,40]. By expanding and collimating the beam, placing attenuation modules along the propagation path, and adjusting the polarization state, a circularly polarized femtosecond laser beam with low power can be generated. The debris adsorption device in the femtosecond laser precision micromachining system is utilized to capture debris produced during the machining process. The ultrafast pulses generated by femtosecond lasers facilitate nonlinear absorption processes, minimizing thermal effects and resulting in precise material removal and modification at the micro- and nanoscale levels [41]. The femtosecond laser has emerged as an ideal tool for SiC machining and is currently attracting growing attention.

In this comprehensive review, we aim to explore the diverse applications and manufacturing aspects of femtosecond laser processing in SiC. Various processing methods such as direct processing, composite processing, processing environment, and beam shaping were explored. Additionally, the advantages and limitations of femtosecond laser processing in SiC were analyzed, along with highlighting recent advancements in the field. By offering an overview of the current state of the art, challenges, and future prospects, this review aims to enhance the understanding of the potential and opportunities presented by femtosecond laser processing in SiC applications. Researchers and engineers in the field stand to gain valuable insights from this review, empowering them to discover new pathways and advance the frontiers of SiC-based technologies.

## 2. Processing Methods

### 2.1. Direct Processing

#### 2.1.1. Conventional Processing Methods

**Figure 2:**
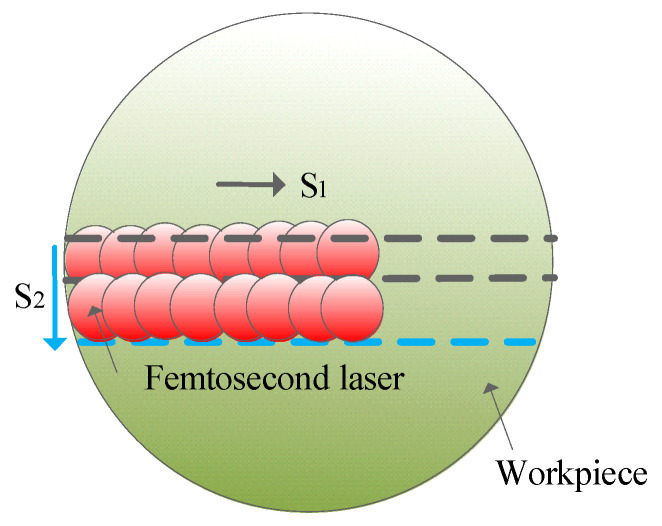
Femtosecond laser direct processing method.

Figure 2 illustrates the femtosecond laser direct processing method. Femtosecond laser direct processing of SiC involves creating different surface features like craters, ripple structures, nanostructures, grooves, and large surface areas.

1. Craters

Numerous scholars have conducted research in this field and have obtained the following results. Percussion drilling is accomplished by focusing femtosecond laser pulses onto the material surface, with each laser pulse removing a specific depth and radius of material. This process gradually increases the depth of the micro-craters, leading to the formation of a micro-crater structure [42]. Percussion drilling demonstrates higher efficiency compared to scanning and helical processing methods. However, when the pulse count reaches a certain value, there may be a termination of processing as the number of pulses increases [43,44]. Additionally, when the pulse count reaches a certain value, the micro-craters may exhibit bending [45,46]. The taper and the width of the heat-affected zone are two quality indicators for craters, typically considered better when smaller in size. The experiment demonstrated that optimizing laser fluence, scan speed, and focal position can lead to achieving good crater quality [47]. When the focal point is above the surface of the workpiece, larger diameter ablation craters can be machined, but a conical structure may result due to laser divergence. Conversely, positioning the focal point below the surface of the workpiece can also create larger diameter ablation craters, but a pointed crater bottom may form due to laser beam contraction incidence [48,49].

**Figure 3:**
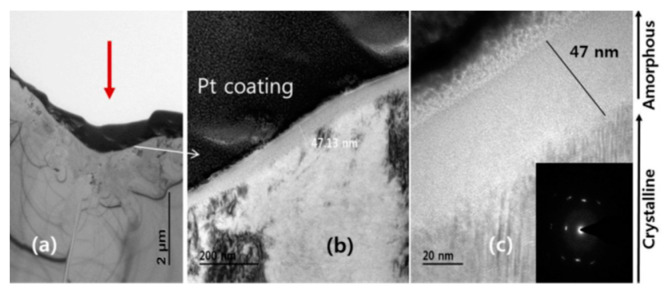
TEM image of femtosecond-laser-modified SiC surface at a peak fluence of 124 J/cm^2^. (**a**) Low resolution view. (**b**) Magnified TEM image indicated by an arrow in (**a**). (**c**) High-resolution image [50].

A comprehensive examination of the surface morphology of SiC following femtosecond laser processing uncovered bond breakage within the carbide ablation craters, the conversion of crystalline SiC to amorphous silicon and amorphous carbon, and the presence of a thin, deformed, and accumulated amorphous layer near the geometric focus of the subsurface in the laser-modified zone [50,51,52]. The TEM image of femtosecond-laser-modified SiC surface at a peak fluence of 124 J/cm^2^ was shown in Figure 3.

Due to the targeted and detailed analysis of the mechanisms involved in the femtosecond laser–SiC interaction in the author’s published papers—Refs. [41,43]—the relevant processes induced by femtosecond laser pulses in SiC will be briefly introduced in this paper. The energy transfer of craters in femtosecond laser processing of single crystal SiC can be described as the main role of the free carrier absorption coefficient in the total absorption coefficient [41]. The primary carrier density absorption mechanism is two-photon absorption, followed by three-photon absorption and Auger recombination effect, with collision ionization playing a less significant role. By integrating THz-TDS scattering near-field optical microscopy with ultrafast pump–probe spectroscopy, the dynamics of photo-excited carriers in semiconductors can be examined at femtosecond and nanoscale resolutions, enabling the observation of carrier decay processes up to 100 ps [53]. The damage threshold energy density and non-thermal melting threshold at a wavelength of 800 nm increase with the pulse width, and the damage threshold energy density is inversely proportional to the wavelength. Both non-thermal melting and thermal melting play a role in material removal through melting [43]. Femtosecond laser drilling on SiC can be categorized into single pulse processing and multi-pulse processing. The material removal mechanism during femtosecond laser single pulse machining of SiC involves Coulomb explosion, photomechanical fragmentation, and thermal melting. These physical mechanisms contribute to material removal under low energy density ablation. On the other hand, non-thermal melting, thermal melting, and vaporization are the primary removal mechanisms at high energy densities. Furthermore, at even higher energy densities, phase explosion, thermal melting, vaporization, and non-thermal melting become the dominant removal mechanisms [43,54]. Vaporization may occur in the form of surface evaporation or explosive decomposition (phase explosion) of superheated liquids [55]. The nanoparticles formed during the phase explosion process and through condensation in the ablation plume are closely linked to the thermodynamic state of the irradiated material and the conditions within the laser ablation plume, primarily driven by thermal processes. Multi-pulse ablation demonstrates incubation effects, where the accumulation of pulse numbers can modify the optical properties of the surface being irradiated and the deposition of laser energy [56,57,58].

2. Ripple Structures, Nanostructures, and Grooves

**Figure 4:**
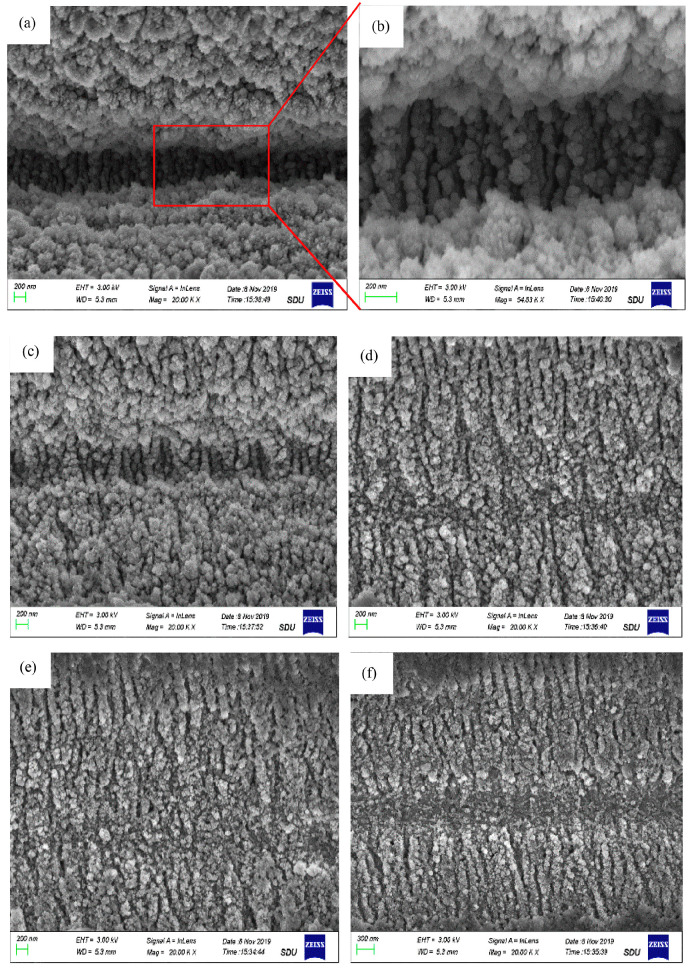
Evolution of surface morphology in laser ablation areas at scan speed S: (**a**,**b**) 50 μm/s, (**c**) 100 μm/s, (**d**) 200 μm/s, (**e**) 300 μm/s, and (**f**) 400 μm/s (fluence, F = 0.76 J/cm^2^) [59].

The evolution of surface morphology in laser ablation areas at different scan speeds is shown in Figure 4. It has shown that as the scan speed decreases, micro/nanostructures can transform from fine ripples, coarse ripples, and nanoparticles into V-shaped grooves. When femtosecond laser lines are applied to SiC, with the increase in energy density and the decrease of scan speed, the processed structure gradually evolves from fine ripples, coarse ripples, and nanoparticles to V-shaped grooves [59,60]. The depth of the groove increases with higher laser energy density, repetition frequency, multi-pass scanning, and numerical aperture. Conversely, it decreases with increased scan speed. The groove width, material removal rate, and heat affected zone increase with higher laser energy density, repetition frequency, and multi-pass scanning but decrease with increased scan speed and numerical aperture. Improving the aspect ratio can be achieved by increasing the number of scans and laser energy density, reducing the scan speed, and minimizing the z-layer feed step [61,62]. The direction of the ripple structure is influenced by the laser polarization angle. For a linearly polarized laser, the ripple structure direction is perpendicular to the laser polarization direction [63,64,65]. A self-organization model based on laser-induced surface instability can be used to explain this phenomenon [66,67,68]. The model suggests that changes in the electric field direction disrupt the surface symmetry, causing the ripple direction to rotate with the laser electric field direction, always remaining perpendicular to the laser electric field direction. For elliptically polarized laser, the ripple structure direction is perpendicular to the major axis of the ellipse, but the continuity of the ripple structure is poorer. For circularly polarized laser, well-defined ripple structures are not observed [63].

3. Large surface areas

Due to the limited processing size of the femtosecond laser on SiC, which is typically confined to a single point or a single line segment, its practical application prospects are restricted. However, by optimizing parameters such as energy density, scan speed, and scanning spacing, it becomes possible to fabricate larger periodic structural arrays on SiC using femtosecond laser processing [51,69]. Under high laser energy density, SiC undergoes selective sublimation, leading to unbalanced removal of carbon and silicon elements. With increasing energy density, the number of hanging bonds increases, along with an enhanced ability to capture oxygen elements [70,71]. Residual tensile stress is generated in the preparation of SiC periodic arrays using a femtosecond laser, and the residual tensile stress increases with the increase in scanning distance. Laser energy density can increase lattice disorder [72,73]. Femtosecond lasers can also be applied to polish SiC [74]. The polishing results of the original surface defects of SiC have a significant impact, and a small incident angle is more conducive to obtaining a smooth surface and significantly reducing the degree of oxidation and graphitization in the ablation zone [74]. Due to the different laser absorption rates and energy distribution of SiC at different laser incidence angles, the ablation threshold and morphology of SiC change with the change in the laser incidence angle [75].

#### 2.1.2. Advanced Processing Methods

1. Processing Environment

Table 1 shows the comparison of processing results under different processing environments. In an air environment, air ionization results in a partial loss of laser energy, leading to a reduction in ablation size and negative effects such as debris eruption [76,77]. In a vacuum environment, the reduction in ambient pressure leads to lower gas molecule density, weakening the ionization effect of air and decreasing laser energy loss. This results in improved surface quality. Conversely, an increase in ambient pressure causes more collisions between plasmas, reducing energy loss and increasing ablation depth [78]. However, this also leads to an increase in the deposition of sediment around the ablation crater. When processing materials in environments with etching gases or nitrogen, it can result in a rough surface quality within the processing area [79]. When comparing corrugated structures processed in air with those generated in an argon environment, the latter shows smaller surface defects and higher ripple heights. X-ray photoelectron spectroscopy analysis reveals a decrease in workpiece surface oxidation during processing in an argon atmosphere [80]. When comparing corrugated structures generated in an argon environment to those processed in air, the former exhibits higher diffraction light intensity, minimal surface defects, and higher amplitude ripples. X-ray photoelectron spectroscopy analysis indicates a reduced ratio of oxygen to metal species on the sample surface in an argon atmosphere, resulting in minimal oxidation [80]. In the case of glass samples subjected to femtosecond laser micromachining in various solutions (including NaCl and KOH aqueous solutions, water, and on the surface of the workpiece), the resulting groove depths and widths were evaluated [81]. At low scanning speeds (50 mm/s), the dissolution of NaCl in distilled water led to an increase in charge density and accelerated multiphoton ionization in the ablation region, resulting in deeper grooves. However, under the same conditions, when NaCl aqueous solution was coated on the surface of the glass sample, the groove depth formed was lower compared to dry samples or those formed in water and KOH aqueous solutions. The study suggests that under multiple scanning glass cutting conditions, the localized temperature increase caused by laser irradiation enhances the corrosion rate of KOH solution, resulting in higher groove depths. The addition of a KOH aqueous solution film improves ablation efficiency, leading to increased groove depth. Furthermore, low-temperature femtosecond laser processing can suppress plasma shielding effects, increase ablation depth, significantly improve surface quality, reduce oxidation and surface roughness, and mitigate thermal accumulation effects [82].

2. Beam Shaping

**Figure 5:**
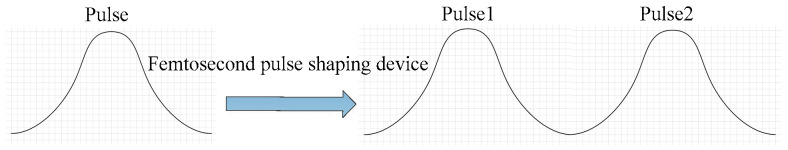
Processing by ultrasonic vibration compounded with femtosecond laser.

**Figure 6:**
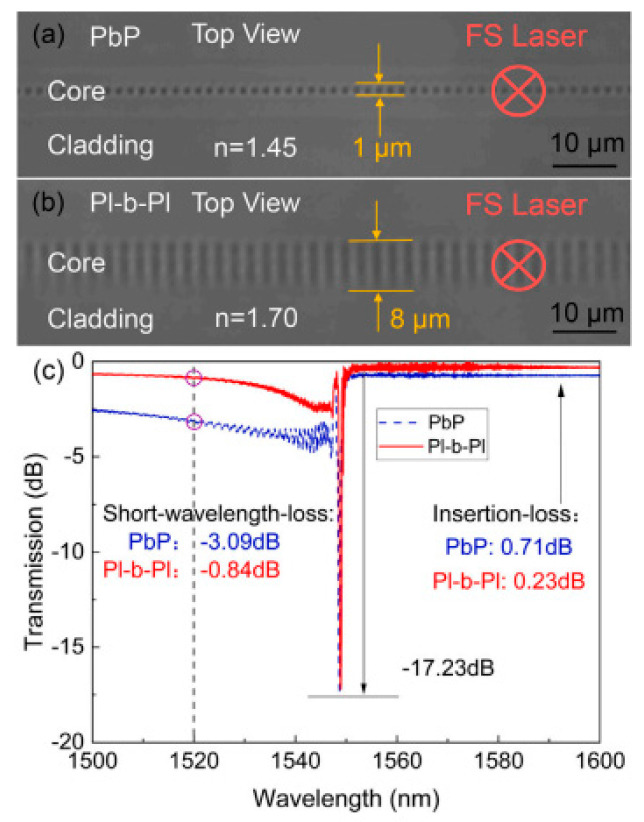
The effects of different types of refractive index oils on the morphology of the grating and its transmission spectra: (**a**) n = 1.45, (**b**) n = 1.7, and (**c**) transmission [93].

Many scholars have also investigated modifying laser beam characteristics to enhance processing outcomes. By utilizing diffraction optical software, the laser beam can be split into several beams, enabling the creation of several modified regions with similar surface morphology in a single scan [83]. In addition to obtaining the same beam of laser in a single scanning process, femtosecond laser pulses can also be divided into several sub pulses that are delayed in time [84]. The delay time of the next sub pulse in two adjacent femtosecond laser sub pulses can be adjusted by the time it reaches the corresponding reflector [85]. The modified regions can then be selectively chemically etched to remove the modified material, resulting in an increase in processing efficiency and consistency. Adjusting parameters like the number of laser beams, beam spacing, and beam energy can optimize the manufacturing of microlens arrays, resulting in superior optical performance [86]. Figure 5 is a schematic representation illustrating the shaping of a single pulse into two pulses. In the process of dual-pulse ablation, when the energy density of the dual pulses is relatively low, the ablation depth decreases as the delay time between the pulses increases [87]. The maximum ablation depth is reached when the delay time is zero, and the ablation depth of the dual pulses is significantly greater than that of a single pulse with the same energy. When the energy density of the dual pulses is high, the delay time has minimal impact on the ablation depth [87]. When the energy of the first pulse is higher than that of the second pulse, the ablation depth noticeably increases, and the recast layer is significantly reduced [88]. Multiphysics layer simulations enable theoretical investigations of the ultrafast thermalization dynamics induced by femtosecond laser double-pulse vortex excitation. Enhanced optical absorption and amplified carrier–phonon coupling dynamics due to double-pulse vortex excitation can further amplify the vortex thermodynamics [89,90]. In addition to temporal shaping, beam shaping can also be performed in space. The Gaussian beam’s longitudinal energy distribution is limited due to its propagation attenuation characteristics, leading to reduced ablation size. In contrast, Bessel beams can maintain a constant profile over long distances [91]. By converting a Gaussian beam into a line beam using a cylindrical lens and adjusting the scanning direction perpendicular to the principal axis, the manufacturing efficiency of anti-reflective structures can be significantly enhanced [92]. Additionally, a new method for high-quality fiber Bragg grating fabrication utilizes high refractive index oil to exploit the transverse dispersion effects along the fiber, altering the focal length of the laser beam without requiring additional spatial beam-shaping devices. This approach allows for flexible control over the width and height of the grating plane [93]. Figure 6 shows the effects of different types of refractive index oils on the morphology of the grating and its transmission spectra.

#### 2.1.3. Existing Problems and Development Trends

Although the surface morphology and size variation of craters, ripple structures, nanostructures, grooves, and large surface areas resulting from femtosecond laser processing of SiC have been extensively studied, the understanding of the processing mechanisms is currently limited to the formation of ablation craters on SiC via femtosecond laser irradiation. There is a lack of research on the mechanism of femtosecond laser processing of SiC when the laser spot moves both laterally and longitudinally. Further research and exploration are needed to study and expand the processing techniques, processing mechanisms, and applications of processing environments and beam shaping technology. Additionally, the Gaussian beam’s longitudinal energy distribution is limited due to its propagation attenuation characteristics, leading to reduced ablation size. In micro-crater machining, it is challenging to generate sufficient intensity of ablation as femtosecond laser pulses are distributed within a specific area.

### 2.2. Composite Processing

#### 2.2.1. Laser–Water Jet Composite Processing Technology

**Figure 7:**
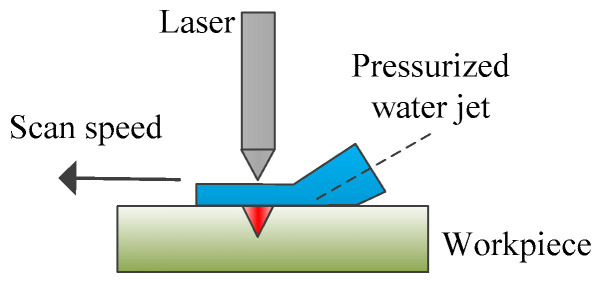
Hybrid laser–water jet processing method.

**Figure 8:**
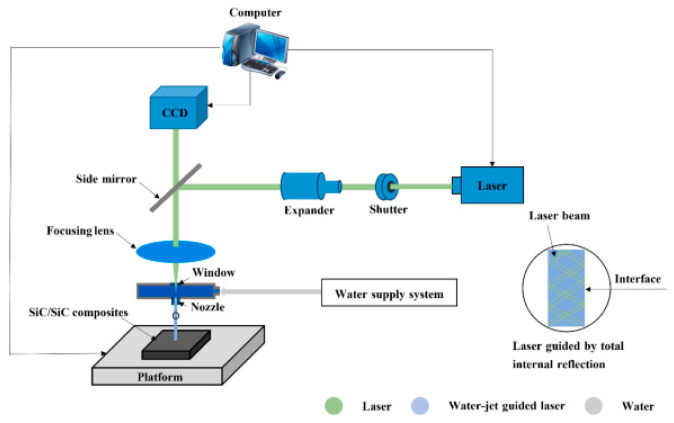
Schematic diagram of laser–water jet equipment [95].

**Figure 9:**
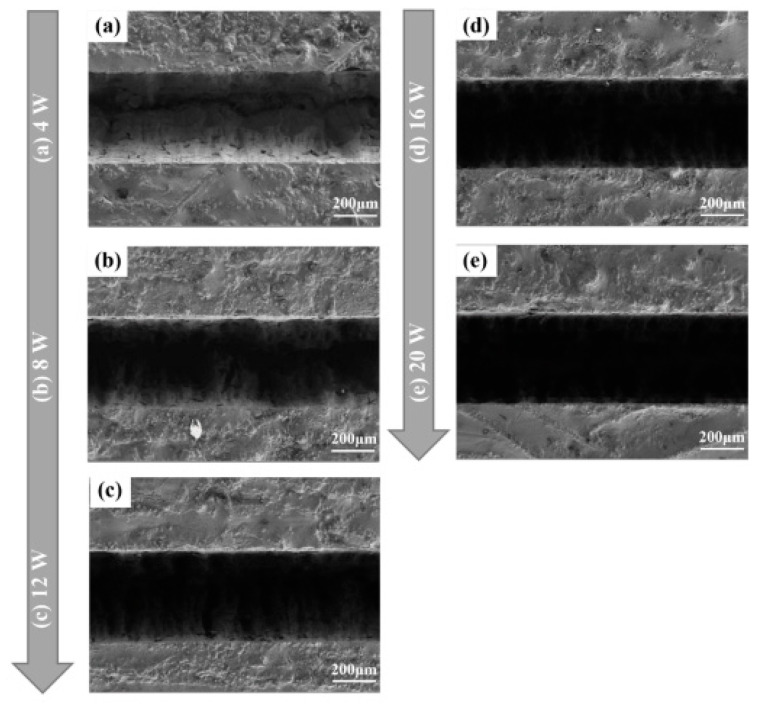
The surface morphology of the micro-groove: (**a**) P = 4 W, (**b**) P = 8 W, (**c**) P = 12 W, (**d**) P = 16 W, and (**e**) P = 20 W [95].

In laser water jet composite processing technology, as depicted in Figure 7, the material is softened through laser heating, which reduces its yield strength. Subsequently, the high-pressure water jet impacts the heated material, causing shear forces and facilitating its removal [9,94]. Figure 8 shows a schematic diagram of laser–water jet equipment. During the laser–water jet machining of SiC, the maximum wall shear stress decreases as the nozzle target distance increases due to the divergence of the water jet. The turbulence and aeration of the high-pressure water lead to a degradation in the quality of the laser beam once it enters the water [9]. Figure 9 shows the surface morphology of the micro-groove. There are no obvious recast layers, cracks, or debris on the edges of the micro-grooves. As the laser power increases, the bottom of the micro-groove gradually darkens until it is no longer visible [95]. To achieve high-precision drilling, Li et al. [96] proposed a method that involves laser direct drilling followed by secondary refinement of micro-crater shapes using coaxial water-jet-assisted laser drilling. Computational fluid dynamics simulations were utilized to optimize the water channel structure and nozzle shape in the device. Furthermore, response surface methodology was employed to optimize multiple process parameters affecting the crater entrance diameter, exit diameter, and taper.

Laser–water jet machining is an advanced processing technology capable of executing complex and precise cuts. It can handle a variety of materials, including those sensitive to high temperatures, as the water jet helps cool the material and prevents heat damage. Unlike traditional mechanical cutting, laser–water jet machining does not require frequent replacement of tools or drill bits, reducing equipment wear. Additionally, this method does not produce harmful gases or significant waste. However, as the energy of the water jet dissipates when penetrating thick materials, it limits the thickness of the materials that can be cut. Compared to pure laser cutting, the processing speed of laser–water jet machining is slower because the water jet takes time to work through the material. The equipment not only has a high initial cost but also consumes a substantial amount of electricity and water during operation. The energy requirements of high-pressure water pumps and laser systems make the overall operational costs relatively high. The operation of high-pressure water jets generates noise and vibration, necessitating additional soundproofing or vibration damping measures. The precision of water jet machining may be affected by the quality of water used. Impurities and hardness in the water can impact the cutting results and normal operation of the machine.

#### 2.2.2. Underwater Laser Composite Processing Technology

**Figure 10:**
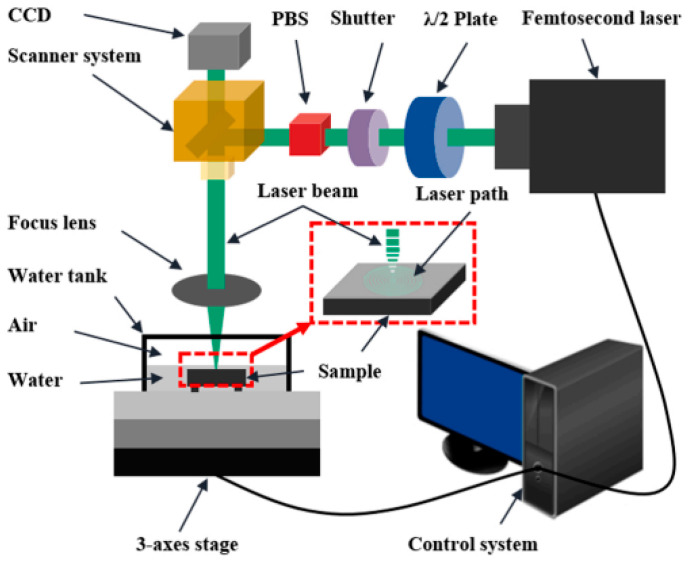
The equipment diagram for underwater processing [104].

**Figure 11:**
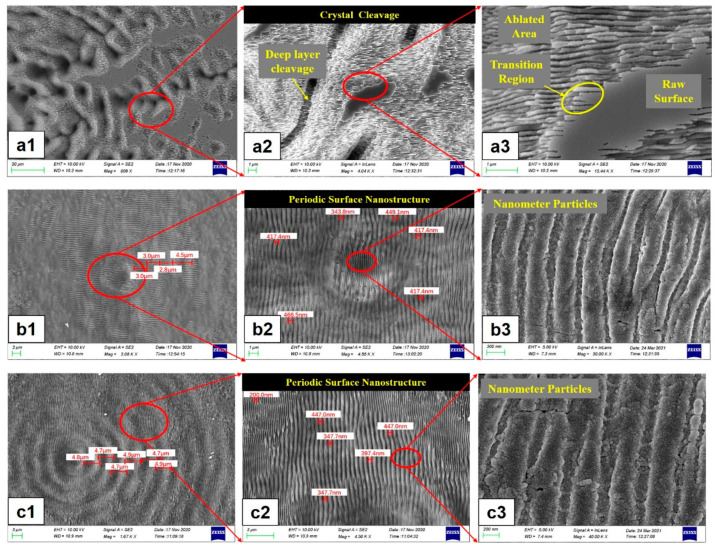
The surface morphology of the craters: (**a1**–**a3**) in air, (**b1**–**b3**) in water, and (**c1**–**c3**) in HF [105].

To mitigate the recast layer and thermal damage associated with higher energy density in direct femtosecond laser processing of SiC, various methods can be employed. Ethanol, distilled water, and underwater processing have been found to reduce re-deposition and thermal damage caused by ablative materials, effectively minimizing oxygen element incorporation [97,98,99]. The assistance of liquid media can help reduce the re-deposition of debris, obtain a smooth sidewall surface, and obtain structures with high material removal rates [100,101]. Ren et al. [102] used an underwater femtosecond laser to process craters in a layered circular pattern by adjusting the focus position. This technology can significantly increase crater depth, reduce adverse thermal effects, surface roughness, and recast layers. It was found that the diameter, taper, wall quality, and surface uniformity of holes are affected by the repetition frequency. In order to eliminate micro-crater defects and achieve consistency in the roundness of the inlet and outlet of the craters, copper or aluminum can be coated on the surface of SiC as a protective layer. The protective layer and water are used to assist the femtosecond laser in processing craters on SiC. After processing, the protective layer is removed via chemical corrosion. In the femtosecond laser experimental equipment, a 2× beam expander was used to obtain a smaller focused spot and to improve the roundness of the hole. The λ/4-wave plate was used to convert linearly polarized light into circularly polarized light. In order to filter out stray light around the laser beam, an aperture was used [103]. The equipment diagram for underwater processing is shown in Figure 10. In the water environment, the inhibition of oxidation reaction occurred via a water film during the decomposition process of SiC [104]. The temperature and oxygen content in the ablation area are the main reasons for corrosion mechanisms in both air and water. During processing in a liquid medium, in addition to the periodic diffraction of plasma in air, high-temperature melting of crystals in the liquid also occurs [105]. Therefore, nanoparticles can be observed in Figure 11. However, the presence of laser-induced bubbles and material deposition introduces uncertainty, which significantly impacts the reflection, refraction, and scattering of laser beams.

#### 2.2.3. Laser–Wet Etching Composite Processing Technology

**Figure 12:**
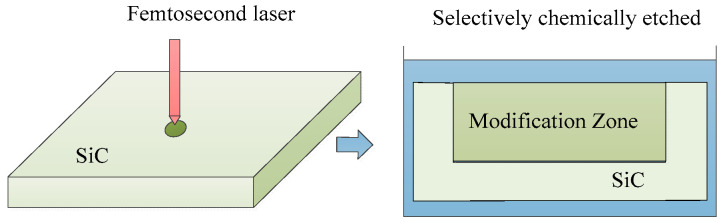
Femtosecond laser combined with selective chemical etching processing method.

**Figure 13:**
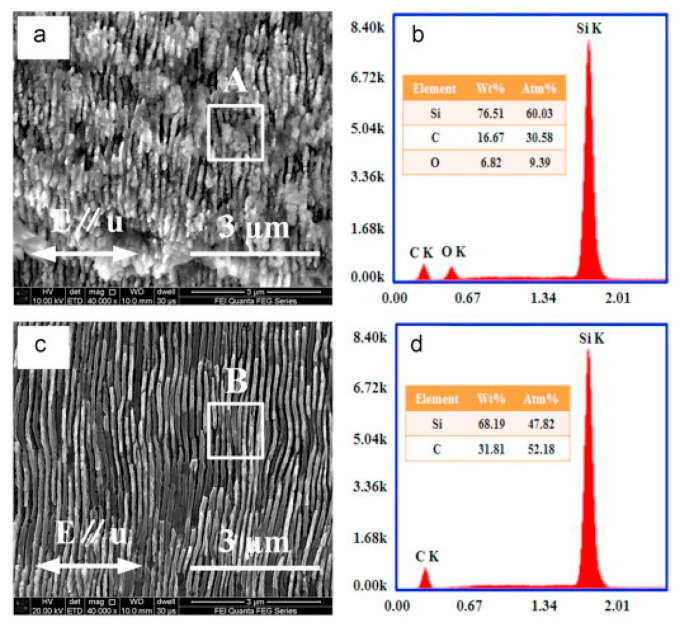
The LISC zone: (**a**) cross-section SEM images before chemical-selective etching; (**b**) chemical composition of area A in (**a**); (**c**) cross-section SEM images after chemical-selective etching; (**d**) chemical composition of area B in (**c**) [108].

Due to the exceptional chemical stability of SiC, it can undergo selective chemical etching after being modified via femtosecond lasers, as illustrated in Figure 12. Following femtosecond laser processing, crystalline SiC transforms into amorphous SiC, which can undergo chemical reactions with a mixture of hydrofluoric acid and nitric acid [106,107,108]. This process enables well-defined and clean SiC ripples without introducing impurities, as shown in Figure 13. Raman spectroscopy can be used to measure the crystallinity of corrugated structures before and after etching. It was found that the Raman intensity of amorphous SiC is highly correlated with the etching rate of the ripple structure [109]. However, when the laser fluence is high, significant ablation occurs on the surface of SiC, resulting in poor surface roughness.

In order to improve the surface quality after etching, wet etching was performed in an ultrasonic cleaning instrument with a heating function. Wang et al. [110] found that the etching rate was related to the crystal direction, and the size of the etching pit was related to the etching time. Wet etching of array structures composed of multiple microcraters, groove array structures, and grid array structures can achieve super hydrophilic performance, a high absorption rate, and low light reflectivity on silicon surfaces.

**Figure 14:**
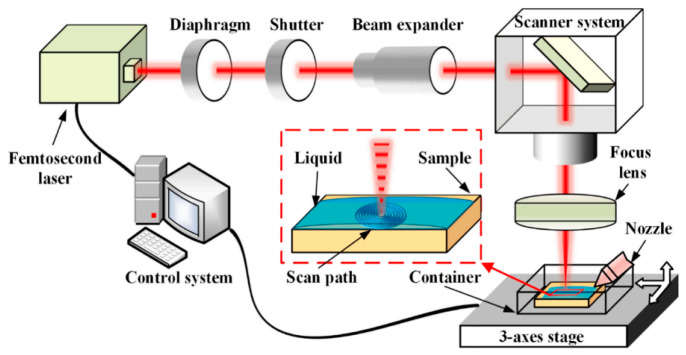
The experimental equipment diagram [113].

In terms of femtosecond-laser-assisted wet etching, other process methods can also be introduced. In order to achieve efficient and high-quality processing, silicon can be processed using a femtosecond laser in air and deionized water, respectively, followed by wet etching, which can improve the optical properties of silicon [111]. A Bessel beam femtosecond laser can be used to assist in wet etching to prepare small taper, large depth, and high aspect ratio structures [112]. This preparation method is simple and can shorten processing time by about 10 times. The combination of femtosecond laser with dynamic wet etching significantly removes the recast layer from the surfaces of micro-craters in SiC/SiC [113]. This is attributed to the SiO_2_ etching induced by NaOH solution in a wet oxygen coupling environment at high temperatures. The experimental equipment diagram is shown in Figure 14. However, the interaction between the laser and the solution causes shock pressure, resulting in ablation pits, fractured SiC fibers, fragmented SiC matrix, and cracks at the bottom of the SiC/SiC holes. Wet etching reactions occur in both the laser-induced oxide layer and localized high-temperature regions. The removal mechanism of SiC/SiC treated with a femtosecond laser combined with dynamic wet etching involves a wet oxygen coupling environment, high-temperature decomposition, localized chemical wet etching, and the influence of the liquid layer.

#### 2.2.4. Laser–Electrochemical Composite Processing Technology

**Figure 15:**
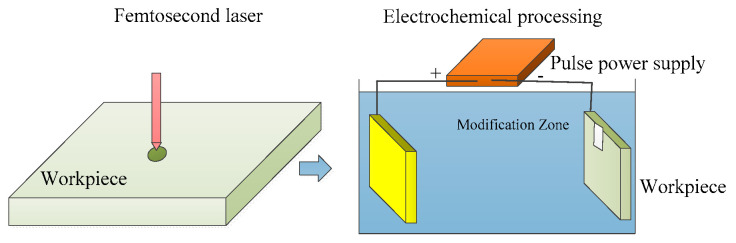
Laser–electrochemical composite processing technology.

**Figure 16:**
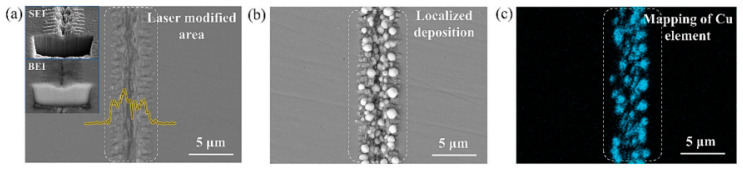
(**a**) Surface morphology diagram of laser-structured steel surface. (**b**) Surface morphology diagram of steel surface after electrochemical deposition. (**c**) Distribution of copper elements in sedimentary areas [114].

Laser–electrochemical composite processing technology combines laser and electrochemical machining processes within single equipment for processing purposes [114]. Figure 15 shows the schematic diagram of laser–electrochemical composite processing technology. The temperature rise caused by the laser can enhance kinetic effects in the electrochemical reaction zone, resulting in an increase in current density. In electrochemical machining conditions that promote the formation of passivating electrolytes, a passive layer forms on the workpiece surface. However, the laser weakens the microstructure of the surface passive layer. Figure 16 shows the morphology of laser electrochemical composite processing. In order to measure the sound pressure signal resulting from pulse laser breakdown of electrolytes and study the influence of cavitation on laser–electrochemical composite processing, a hydrophone was employed to capture the sound pressure signal generated when a pulsed laser was focused on the electrolyte [115]. A detection system for laser–electrochemical composite processing was established for this purpose. During laser and electrochemical composite etching, impact cavitation plays a vital role in material removal. As the laser energy increases, both the bubble radius and energy expand. Higher laser energy leads to the formation of plasma shock waves and jet forces, resulting in more efficient material removal. Bubble pulsation facilitates electrolyte flow, ensuring a constant supply of fresh ions and enhancing the electrochemical reaction rate. Moreover, it reduces the heat-affected zone and improves the surface quality of microstructure etching. The combination of laser processing and electrochemical etching creates micro-grooves on the material surface, which helps reduce friction with the surroundings. The width and density of these grooves are crucial in determining the friction reduction effect. Initially, as the width and density of the grooves increase, more contact points form between the material and the external medium, leading to an increase in the surface friction coefficient. However, beyond a certain point, further widening or increasing the density of the grooves may cause a decrease in the surface friction coefficient due to the reduced effective contact area [116]. In laser–electrolytic composite machining, the system incorporates a plunger hydraulic pump and an electric control valve to pressurize the electrolyte and regulate its flow rate. Temperature sensors and ion selective electrodes are utilized for data detection, enabling real-time control of electric control valves, heaters, and other components. This ensures automatic control of the electrolyte circulation control system [117]. In the synchronous processing of jet-assisted laser and electrochemical composite materials, the laser is focused on the processing area through an electrolyte jet. The metal nozzle serves as the cathode, while the workpiece acts as the anode [118]. Synchronous jet-assisted laser and electrochemical composite machining can achieve electrochemical dissolution of workpiece materials, simultaneously remove the recast layer on the laser-processed surface, and reduce thermal impact. It improves mass transfer efficiency in the processing area, enhances electrochemical processing efficiency, reduces laser energy loss caused by processing products, and increases the depth of composite processing. The method also reduces surface sputtering during laser processing, provides timely cooling to the laser processing area, and minimizes thermal impact. Laser–electrolytic sequential composite machining eliminates the need for a laser stable coupling device. The material removal mechanism in each individual process is well studied, and electrolytic machining improves surface quality while allowing control over machining accuracy. However, challenges arise in the process of laser–electrolytic sequential composite machining. The processing head requires frequent replacement, and accurate positioning of the wire or tube-shaped micro tool electrode at the center of the laser-preformed hole is crucial. Misalignment between the tool electrode and the laser preformed hole, as well as uneven distribution of the recast layer from laser processing, can result in variations in the electrolysis rate of the crater wall and fluctuations in the crater wall itself. This makes it difficult to maintain precision consistency in micro-crater processing. Moreover, the technology has limitations in deep processing due to the restricted depth of laser drilling [118]. However, the laser and electrochemical composite machining process is complex, involving the intricate coupling of multiple physical fields such as the light field, flow field, electrochemical dissolution field, thermal field, etc. The distribution patterns of processing products, bubbles, plasma, and other phenomena within the machining gap are not yet well understood, making it challenging to model the laser and electrochemical composite machining process accurately. Additionally, some experimental process parameter data are difficult to monitor. Therefore, modeling and simulation optimization of the process are crucial for providing support in intelligent control of the machining process, enhancing its controllability, and improving predictability.

#### 2.2.5. Laser–Ultrasonic Vibration Composite Processing Technology

**Figure 17:**
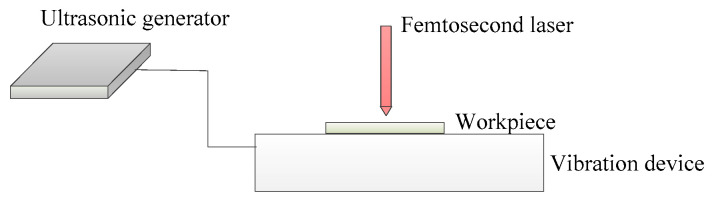
Processing via ultrasonic vibration compounded with femtosecond laser.

**Figure 18:**
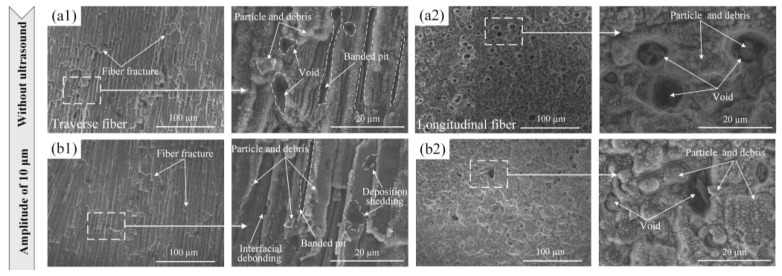
Surface morphology of C/SiC composites processed at different ultrasonic amplitudes: (**a1**,**a2**) without ultrasound; (**b1**,**b2**) 10 µm [120].

By combining ultrasonic vibration with ultrafast laser technology, the efficiency of ultrafast laser micro-crater manufacturing can be further enhanced. This combination improves the depth-to-diameter ratio of micro-craters and enhances the quality of micro-crater morphology [119]. Figure 17 shows the schematic diagram of ultrasonic vibration compounded with femtosecond laser composite processing technology. Figure 18 depicts the surface morphology of C/SiC composite materials processed under different ultrasonic amplitudes. Compared to femtosecond laser polishing alone, ultrasonic-vibration-assisted femtosecond laser polishing enhances the material removal rate and reduces particles, debris, and surface oxidation, while also increasing residual compressive stress in carbon fibers [120]. Currently, there are two main forms of ultrasonic-vibration-assisted laser drilling: one involves applying ultrasonic vibration to the laser lens, while the other involves applying ultrasonic vibration directly to the workpiece. When ultrasonic vibration acts on the laser lens, it can be controlled to change the energy distribution at the laser focal point, resulting in better processing quality of micro holes compared to traditional laser drilling methods. Ultrasonic vibration applied to the workpiece effectively enhances the absorption of the laser beam via the material inside the hole, further promoting beam reflection and improving the processing depth and efficiency of micro holes. When vertical ultrasonic vibration and coaxial low-pressure assistance are used, additional kinetic energy is provided for the flow and injection of the molten material, enhancing the material removal rate. This approach also reduces re-solidification of splashed melt on the crater wall and decreases the thickness of the recast layer. During laser processing, the material undergoes heating, melting, and evaporation. The sound pressure gradient generated via ultrasonic vibration within the molten pool promotes continuous circulation and flow of the molten material. Additionally, high-frequency ultrasonic vibration facilitates the flow of plasma above the processing area, which helps improve the quality of micro-crater processing. The mixing and stirring effects induced by ultrasound enhance the flow and convection of molten materials within the pores. This improves the homogenization of composition and microstructure, reduces component segregation, and minimizes the formation of micro-cracks [121]. The composite processing technology that combines ultrasonic vibration, laser processing, and water jet processing incorporates the advantages of these three methods [122]. In this technology, the ultrasonic module utilizes a direct connection between the ultrasonic transducer, vibration plate (where the workpiece is placed), and the multifunctional, ultra-precision fine tuning platform. This setup eliminates system errors caused by laser vibration and enhances the stability of the processing process. The amplitude is measured using a laser displacement sensor. The jet system is powered by an electric-motor-driven plunger pump, and the jet parameters are controlled by adjusting the overflow valve and throttle valve [122]. By incorporating ultrasonic vibration into the process using the same laser parameters, the depth of laser-etched grooves gradually increases in conjunction with an elevation in laser current. The cavitation effect induced by high-frequency ultrasonic vibration enhances the kinetic energy of the molten pool and promotes vertical agitation of the slag. During laser cutting, part of the molten material evaporates, while another part is removed from the ablation groove due to the impact of the water jet. The combination of ultrasonic water jet and laser processing significantly reduces the surface recast layer in the groove. Additionally, there is a small amount of crystallization at the groove boundaries, and the groove width increases, resulting in relatively good surface quality. However, due to increased energy loss caused by the water jet’s impact during laser etching, the groove depth decreases.

#### 2.2.6. Laser–Chemical Mechanical Polishing Processing Technology

The femtosecond laser–chemical mechanical polishing (CMP) hybrid processing technique involves the initial use of a femtosecond laser for material pre-treatment, followed by the application of colloidal silica slurry to polish the workpiece surface under a polishing pressure of 120 kPa and a rotational speed of 50 min^−1^ [123]. The ripple structures generated on the workpiece surface by the femtosecond laser facilitate increased the contact area between the CMP slurry and the substrate surface. Processing in ambient air introduces oxygen elements into the laser-modified zone and generates an amorphous layer, both of which enhance the CMP process [124]. In the femtosecond laser–CMP hybrid processing technique, the material removal rates of the C-face and Si-face of silicon carbide samples increased by 77% and 207%, respectively, compared to untreated samples [125]. Additionally, the research found that the surface roughness (Ra) of the workpiece polished with alumina slurry was reduced to as low as 0.081 nm [126]. However, the oxide coating on the SiC surface reduced the likelihood of abrasive contact with the SiC-Si surface, resulting in minor scratching after polishing.

The femtosecond laser–ultrasonic vibration polishing hybrid method can be used to improve micro-cracks generated during femtosecond laser processing [127]. Diamond particles move up and down at an extremely high speed in the vertical direction relative to the workpiece. In the laser-modified zone, the raised peaks fracture under the intense collision of the diamond particles and are carried away by the diamond slurry, resulting in lower surface roughness.

However, further research is needed to assess the processing efficiency of femtosecond-laser-assisted chemical–mechanical polishing, as well as the evaluation of subsurface stress layers and crystal defects post-processing.

#### 2.2.7. Laser–Inductively Coupled Plasma Etching

The femtosecond laser and inductively coupled plasma (ICP) etching hybrid processing technology can accelerate the etching rate of silicon carbide and achieve a better quality processed surface [128]. Following femtosecond laser irradiation, the ICP etching rate of silicon carbide can be increased by up to 117.18% compared to processing without femtosecond laser treatment [128]. The induced silica and rough surface on silicon carbide via femtosecond-laser-facilitated ICP etching is effectively removed after etching [129]. The femtosecond laser and inductively coupled plasma etching hybrid processing technology can significantly improve the surface morphology at the bottom of the modified area, reduce surface roughness, eliminate amorphous carbon, and enhance its deformation capability.

Laser–inductively coupled plasma etching is an advanced micro- and nano-fabrication technique known for its high precision and efficiency. It enables precise machining at the micro- and even nano-meter scale, boasting exceptional machining resolution and the capability to handle intricate structures and multi-layered devices. Compared to conventional plasma etching techniques, it offers higher processing rates and enhanced production efficiency while minimizing device damage during processing. However, the acquisition and maintenance costs of this equipment are relatively high, and the operation and optimization of process parameters can be complex. Moreover, it demands stringent environmental conditions for operation.

#### 2.2.8. Existing Problems and Development Trends

Femtosecond laser composite processing involves multiple machining processes, necessitating precise control and optimization of various process parameters. Femtosecond laser composite processing systems typically require multiple devices and complex control systems, resulting in high costs. The mechanism of femtosecond laser composite processing remains to be further investigated, and the composite interaction field between femtosecond laser and other processing methods needs to be further determined. Additionally, the surface characteristics after femtosecond laser composite processing need to be further established. Future advancements in laser composite processing will move towards intelligence and automation, leveraging machine learning and artificial intelligence technologies to optimize machining processes and parameter control, thereby enhancing production efficiency and quality stability. The integration of multiple machining methods into a single system will enable multifunctional machining to meet diverse material and processing requirements. Enhancing adaptability and flexibility will be emphasized for different types of materials to achieve a wider range of applications. Furthermore, in order to improve processing efficiency, achieve clean manufacturing, enhance surface quality, and ensure sidewall inclination, new approaches for femtosecond laser composite processing need to be further studied.

## 3. Applications

Depending on the structure of femtosecond laser processing, the processed surface exhibits different applications. Femtosecond laser machining of SiC micro-grooves is primarily used in applications such as micro-cavities, micro-sensors, and micro-mechanical systems. Micro-groove texturing on non-contact single crystal SiC mechanical sealing surfaces can reduce the friction coefficient; improve lubricant flowability; store a certain amount of lubricant, thereby reducing friction; and expanding the range of lubrication applications [130]. Micro-grooves on SiC ceramic surfaces can reduce friction losses in rolling bearings and improve rotational performance [131]. Femtosecond-laser-machined SiC micro-grooves are also commonly applied in droplet-based microfluidic devices [132], microfluidic sensors [133], and biosensors [134], where high aspect ratio micro-grooves help reduce friction in microfluidic devices and improve flow properties [135]. The micro–nano structural morphology induced by femtosecond lasers has potential applications in various fields, including bio-molecular labeling [136], surface texturing [137], self-cleaning surfaces and hydrophobic materials [138], super-wetting surfaces [139], wide-field imaging [140], improved cutting performance [141], solar cells [142], enhanced conductivity of laser-modified regions [143], light absorption enhancement, photocurrent enhancement [144], and MEMS [145]. The structure of SiC can be further extended using femtosecond lasers to create a series of parallel micro-grooves on the surface of SiC. By utilizing the highly graded surface capillary structure of the micro-grooves, water has a very high diffusion function in the inertial capillary flow state, resulting in excellent micro core and nano core suction functions and cooling performance on the surface of SiC, and this function has long-term stability [146]. Therefore, it can be applied to M-cycle air conditioning systems to reduce energy loss, and can also be applied to improve power generation efficiency, waste heat recovery, and thermal management [146]. In the final analysis, the fabrication of micro–nano structures on SiC holds significant practical application significance.

### 3.1. Microelectromechanical Systems

SiC is highly suitable for applications in microelectromechanical systems due to its high mechanical strength, chemical inertness, and electrical stability. It finds uses in actuators, pressure sensors, temperature sensors, transverse resonance devices, optoelectronics, and sensors [147,148]. For large-sized manufactured SiC structures, many scholars are currently committed to applying this structure to sensors. Due to its excellent high-temperature stability, chemical inertness, and mechanical and electrical properties, SiC is an ideal material to replace silicon for manufacturing high-temperature pressure sensors operating at temperatures above 500 °C [149]. Micro-sensing devices made of SiC can achieve remote sensing predictions of high temperatures and maintain high sensitivity [150]. However, SiC is a typical difficult-to-machine material with high hardness and chemical inertness, making it challenging to meet the manufacturing requirements of sensitive diaphragms for high-temperature pressure sensors using traditional mechanical machining and chemical etching methods.

To meet the performance requirements of sensors, femtosecond laser can be utilized for deep etching on SiC diaphragms [151,152]. The sensitivity of pressure sensors manufactured using femtosecond lasers is twice that of other SiC sensors [153]. Femtosecond lasers can be used to fabricate square-shaped sensitive diaphragms on SiC, which exhibit good output characteristics at high and low temperatures [153,154]. Femtosecond lasers can also be used to fabricate circular sensitive diaphragms on SiC, which exhibit good operational capabilities under high temperatures and pressures [155,156,157]. A femtosecond laser was employed to create blind holes with a diameter of 1200 μm and a depth of 270 μm on a 350 μm thick 4H-SiC wafer to form an 80 μm thick circular membrane as the pressure sensor’s diaphragm [151]. The excess SiC material was gradually removed layer by layer using the femtosecond laser until the desired depth of the blind hole was achieved, with the remaining membrane at the bottom serving as the diaphragm for the pressure sensor. The smoothness, flatness, sidewall inclination angle, and surface roughness of the diaphragm are important factors that influence the performance of the sensor [158,159,160]. The sensor was connected to a single-arm bridge for pressure sensor characterization experiments. As pressure was applied and released, the output voltage of the sensor varied accordingly. The nonlinearity error can reach up to 0.13%, and the repeatability error is 1.49% [161,162]. Under extreme temperatures and corrosive environments, the sensitivity of the sensor can reach 76.0 μV/V/MPa, with an average daily drift of only 1.61% [162].

Femtosecond lasers can be used to fabricate accelerometers on SiC [163]. Compared to pressure sensors, accelerometers have a more complex structure and require higher machining precision. To ensure the sensor’s shock resistance and longevity, the morphology of the sensitive beam also needs to meet high standards.

Compared to other machining methods for SiC sensor membranes, using a combination of femtosecond lasers and inductively coupled ICP etching has several advantages, including high machining dimensional accuracy, low surface roughness, and relatively high processing efficiency [164]. A detailed simulation and optimization analysis of sensor size was conducted based on the anisotropic elastic modulus parameters of SiC. Each layer scan differs by 30° from the previous scan path to ensure uniformity of surface removal [165]. After femtosecond laser machining, the processed SiC can undergo wet cleaning and ICP etching to significantly improve the surface morphology of the membrane’s bottom side.

### 3.2. Existing Problems and Development Trends

However, the efficiency of femtosecond laser deep etching on SiC is relatively low, processing quality is difficult to ensure, elliptical and excessive etching are prone to occur, and it is challenging to ensure sidewall inclination. Further research is needed to understand the mechanism of large-area removal of SiC using femtosecond lasers. The next step would involve further exploration and research into efficient and high-quality methods for removing SiC, as well as understanding the underlying mechanisms. The specific applications of femtosecond laser processing on SiC can be further expanded based on the needs of businesses and industries.

## 4. Conclusions

This article provides a detailed discussion on the machining methods and applications of femtosecond laser processing on SiC. The methods for femtosecond laser processing on SiC are categorized into direct processing, hybrid processing, processing environment modification, and beam shaping methods. The application of femtosecond laser processing on SiC in MEMS is also described.

Femtosecond lasers can directly process craters, ripple structures, nanoscale structures, grooves, and large-area surfaces on SiC. After femtosecond laser processing of SiC, the unbalanced removal of silicon and carbon elements in SiC occurs, and oxygen is introduced due to the capturing effect of dangling bonds. The region modified via femtosecond lasers exhibits residual tensile stress and strain, with the residual tensile stress increasing with the scan spacing increment. The modified region also exhibits lattice disorder, which increases with the increase in energy density. During femtosecond laser ablation of SiC, both thermal melting and non-thermal melting coexist. The dominant mechanism for carrier absorption is multiphoton absorption, while collisional ionization has negligible effects.

Laser–water jet hybrid processing, underwater processing, and femtosecond laser and chemical selective etching hybrid processing techniques can be used for SiC machining. Hybrid laser processing methods can effectively increase the ablation depth and reduce the re-deposition of ablated material. However, laser–water jet and underwater processing techniques may affect the beam quality due to fluid turbulence and bubbles. Selective chemical etching using a mixture of hydrofluoric acid and nitric acid is not easy to handle in practical experiments and carries certain risks. Processing in a vacuum environment can reduce energy losses and achieve better surface quality. Beam shaping can be used to obtain uniform processing dimensions or larger aspect ratios, resulting in better surface quality of the material.

Femtosecond laser processing on SiC offers various benefits for the fabrication of sensors, micro-cavities, and micro-mechanical systems. Micro-groove texturing on SiC surfaces offers benefits like reduced friction coefficient, improved lubricant flowability, and enhanced rotational performance. Femtosecond laser technology enables the precise fabrication of highly accurate diaphragms for sensors, including pressure and accelerometer devices. These diaphragms can be created in square or circular shapes with exceptional precision. They exhibit excellent output characteristics even under challenging conditions, such as high temperatures, pressures, and extreme environments. However, challenges remain in terms of low efficiency and sidewall inclination during deep etching, necessitating further research to develop more efficient and high-quality removal methods and mechanisms. Additionally, exploring specific applications of femtosecond laser processing on SiC can be tailored to meet the needs of different industries. An alternative approach using a combination of femtosecond laser and ICP etching shows promise in achieving high machining accuracy and low surface roughness for sensor membranes. Further improvements have been observed through post-processing steps like wet cleaning and ICP etching, significantly enhancing the surface morphology of the processed SiC.

In summary, this review emphasizes on different femtosecond laser composite processing methods and the application of SiC structures processed by femtosecond laser in MEMS. And it is expected to promote the development and application of femtosecond laser composite processing technology.

Despite the contributions of femtosecond laser processing, there are still limitations that need to be addressed in the context of SiC processing. The processing mechanisms for micro-grooving or milling of SiC using femtosecond laser are not yet clear and require further investigation. The mechanisms of femtosecond laser composite processing also need to be studied in more depth. Surface characteristics after femtosecond laser composite processing are still subject to further research. Additionally, new methods for femtosecond laser composite material processing need to be explored to improve processing efficiency, achieve clean manufacturing, enhance surface quality, and ensure sidewall taper. The effects of processing environment and beam shaping on composite processing can be further investigated in the future. Furthermore, the application scope of femtosecond-laser-processed SiC structures needs to be expanded, and process optimization should be performed based on specific application requirements.

## Figures and Tables

**Table 1:** Comparison of processing results in different processing environments.

Processing Environments	Advantage	Disadvantage
Air		Partial loss of laser energy
Vacuum	Weakens the ionization effect of air and reduce laser energy loss	
Nitrogen		Rough surface quality
Argon		Small surface defects and high ripple heights
KOH aqueous solutions	Deep grooves	
Low-temperature	Increases ablation depth, improves surface quality, and mitigates thermal accumulation effects	
